# Corrigendum: Surgical decision-making following neoadjuvant immunotherapy for dMMR rectal cancer; case reports and review of the literature

**DOI:** 10.2340/1651-226X.2024.40158

**Published:** 2024-02-29

This corrigendum lists the corrections for Letter to the Editor titled, *Surgical decision-making following neoadjuvant immunotherapy for dMMR rectal cancer; case reports and review of the literature*, written by Mef Nilbert, Jakob Eberhard, Jenny Engdahl Severin, Marcus Edelhamre, Filip Torle, Dejan Korkocic & Fredrik Jörgren (2023). Published in Acta Oncologica, Volume 62, NO.12, 1945–1951, DOI: https://doi.org/10.1080/0284186X.2023.2281005

**Table:** 

	Location	Error	Correction
1.	p. 1946, table 1, row 20, column ‘Treatment’	Treatment ‘Nivolumab’	Nivolumab has been replaced with ‘Pembrolizumab’
2.	p. 1946, table 1, row 21, column ‘Treatment’	Treatment ‘Nivolumab’	Nivolumab has been replaced with ‘Pembrolizumab’

Corrected table:

**Table 1:** Summary of patients with dMMR rectal cancer reported with neoadjuvant immunotherapy.

Age/sex	Lynch syndrome	Tumor Stage	Mesorectal fascia involved	Treatment	Treatment cycles	Radiologic response	Definitive s surgery	Pathologic response	Clinical response	Reference
27/male	yes	T4bN2º	Yes	Nivolumab (3 mg/kg)	6	CR	yes	ypT0N0	CR	Zhang et al. 2017
62/female	yes	T4bN2º	No	#Nivolumab (3 mg/kg)	6	CR	no	–	CR	Zhang et al. 2017
27/female	yes	T3N2º	Yes	Ipilimumab (1 mg/kg) and nivolumab (3 mg/kg)	2	CR	yes	ypT0N0	CR	Mans et al. 2020
				followed by nivolumab	1					
81/male	NA	T3cN1/T1-2N0	Yes	Pembrolizumab (200 mg)	11	CR	no	–	CR	Demisse et al. 2020
55/male	yes	T3cN1	Yes	*Ipilimumab (1 mg/kg) and nivolumab (3 mg/kg)	3	CR	no	–	CR	Demisse et al. 2020
				followed by maintenancewith nivolumab	7					
38/female	yes	T3N2M0	Yes	FOLFOX combined with pembrolizumab (200 mg)	7	CR	yes	ypT0N0	CR	Demisse et al. 2020
33/male	yes	T3N2º	Yes	Ipilimumab (1 mg/kg) and nivolumab (3 mg/kg)	2	CR	yes	ypT0N0	CR	Trojan et al. 2021
38/female	yes	T4Nþ	NA	Dostarlimab (500 mg every 3 weeks)	9	CR	no	–	CR	Cercek et al. 2022
30/female	yes	T3Nþ	NA	Dostarlimab (500 mg every 3 weeks)	9	CR	no	–	CR	Cercek et al. 2022
61/female	no	T1/T2Nþ	NA	Dostarlimab (500 mg every 3 weeks)	9	CR	no	–	CR	Cercek et al. 2022
28/female	no	T4Nþ	NA	Dostarlimab (500 mg every 3 weeks)	9	CR	no	–	CR	Cercek et al. 2022
53/female	yes	T1/T2Nþ	NA	Dostarlimab (500 mg every 3 weeks)	9	CR	no	–	CR	Cercek et al. 2022
77/female	yes	T1/T2Nþ	NA	Dostarlimab (500 mg every 3 weeks)	9	CR	no	–	CR	Cercek et al. 2022
77/female	no	T1/T2Nþ	NA	Dostarlimab (500 mg every 3 weeks)	9	CR	no	–	CR	Cercek et al. 2022
55/female	yes	T3Nþ	NA	Dostarlimab (500 mg every 3 weeks)	9	CR	no	–	CR	Cercek et al. 2022
68/male	no	T3Nþ	NA	Dostarlimab (500 mg every 3 weeks)	9	CR	no	–	CR	Cercek et al. 2022
78/female	no	T3N0	NA	Dostarlimab (500 mg every 3 weeks)	9	CR	no	–	CR	Cercek et al. 2022
55/female	yes	T3Nþ	NA	Dostarlimab (500 mg every 3 weeks)	9	CR	no	–	CR	Cercek et al. 2022
27/male	no	T3Nþ	NA	Dostarlimab (500 mg every 3 weeks)	9	CR	no	–	CR	Cercek et al. 2022
68/male	yes	T2N0	No	Pembrolizumab (200 mg every 3 weeks)	10	CR	yes	ypT0N0	CR	Present study
60/female	no	T3bN2º	Yes	Pembrolizumab (200 mg every 3 weeks)	11	CR	yes	ypT0N0	CR	Present study

a Lateral lymph node involvement observed.

b Patient treated with neoadjuvant chemoradiotherapy without response prior to immunotherapy.

